# Leveraging Uncertainties in Softmax Decision-Making Models for Low-Power IoT Devices

**DOI:** 10.3390/s20164603

**Published:** 2020-08-16

**Authors:** Chiwoo Cho, Wooyeol Choi, Taewoon Kim

**Affiliations:** 1 Hallym Institute for Data Science and Artificial Intelligence, Hallym University, Chuncheon 24252, Korea; cwcho@hallym.ac.kr; 2 Department of Computer Engineering, Chosun University, Gwangju 61452, Korea; wyc@chosun.ac.kr; 3 School of Software, Hallym University, Chuncheon 24252, Korea

**Keywords:** deep learning, softmax, decision-making, classification, sensor data, Internet of Things

## Abstract

Internet of Things (IoT) devices bring us rich sensor data, such as images capturing the environment. One prominent approach to understanding and utilizing such data is image classification which can be effectively solved by deep learning (DL). Combined with cross-entropy loss, softmax has been widely used for classification problems, despite its limitations. Many efforts have been made to enhance the performance of softmax decision-making models. However, they require complex computations and/or re-training the model, which is computationally prohibited on low-power IoT devices. In this paper, we propose a light-weight framework to enhance the performance of softmax decision-making models for DL. The proposed framework operates with a pre-trained DL model using softmax, without requiring any modification to the model. First, it computes the level of uncertainty as to the model’s prediction, with which misclassified samples are detected. Then, it makes a probabilistic control decision to enhance the decision performance of the given model. We validated the proposed framework by conducting an experiment for IoT car control. The proposed model successfully reduced the control decision errors by up to 96.77% compared to the given DL model, and that suggests the feasibility of building DL-based IoT applications with high accuracy and low complexity.

## 1. Introduction

The unprecedented success of the Internet of Things (IoT) paradigm has changed and reshaped our daily lives. We start a day by asking smart speakers, such as Amazon Echo or Google Home, about the weather, remotely control the home appliances and door lock, and switch off the light from mobile devices before going to bed. As was inevitable, IoT is now playing an important role in many other fields, such as industry [1,2], healthcare [3,4], energy [5,6], transportation [7,8] and environment monitoring [9], to name a few. The increasing number of pervasive and widespread, Internet-connected IoT devices capture the environment and generate an enormous amount of data, which is becoming one of the major sources of information nowadays. To understand such massive sensor data, and thus, to draw meaningful information out of it in an autonomous manner, various approaches have been applied, including deep learning.

Deep learning is a type of or a class of techniques in machine learning [10,11] that surpasses the capacity of machine learning in many applications such as computer vision and pattern recognition. Deep learning is a representation learning technique, and is capable of learning a proper representation for the given task, such as classification or detection, from the sensor data. Deep learning is now one of the most actively studied areas, and it is expected to contribute much to the success of many IoT applications. As a result, applying different deep learning techniques to IoT applications [12,13] is also gaining much attention nowadays.

Among those areas deep learning excels in, we focus on a classification task in this paper. Classification is a popular supervised learning task in deep learning [10], wherein a trained model is asked to predict which of the classes or categories the unseen input belongs to. Deep learning is carried out by using a neural network model that learns features from the input data. For clarification, a deep neural network with a multi-layer, fully connected perceptron is referred to as artificial neural network (ANN), whereas a network with convolution and pooling layers is a convolutional neural network (CNN). For the output classifier of ANN, CNN and other deep neural network models, the softmax function is the most widely used [10] combined with cross-entropy loss, thereby called, softmax loss.

Although the primitive or basic deep neural network models have shown surprisingly excellent classification performances in many applications, many research communities have been striving for better performance. Such efforts can be roughly divided into two research directions. One is to design sophisticated network models [14,15,16], and the other is to replace the softmax loss with an advanced equivalent [17,18,19]. Among these, we briefly review the latter which are closer to what we focus on in this paper.

In general, to achieve a better image recognition performance for face identification and verification, for example, many researchers have focused on how to effectively classify a given face, i.e., mapping a face to a known identity, and determining if the two given faces are identical, respectively [20,21]. For such tasks, the chosen features from the decision boundary can immensely affect the classification or recognition performance. Therefore, extracting ideal features having small intra-class and large inter-class distances under a given metric space is the most important, yet challenging, task. Among the previous work tackling the issue with softmax loss, angular softmax loss for CNN [17] is proposed to adjust the angular margin when the model determines decision boundaries for the feature distribution, and it helps CNN learn angularly discriminative features. In [19], the authors introduced another softmax loss that contributes to incorporating the margin in more instinctively and interpretable manners, while minimizing the intra-class variance, which was considered to be the main drawback of the widely-used softmax loss. Liu et al. [18] proposed a large-margin softmax loss function, called L-softmax, which effectively enhances both intra-class compactness and inter-class separability between the already-learned features. Furthermore, L-softmax can adjust the desired margin, while preventing the network model from being overfitted. With respect to the hardware-centric research, Wang et al. [22] proposed optimizing the softmax function by mitigating its complexity by means of an advanced hardware implementation. The authors showed that by reducing the total number of operations in the constant multiplication, the adjusted softmax function architecture enables multiple algorithm reduction, fast addition and less memory consumption at the expense of a negligible accuracy loss. These approaches successfully enhanced the classification performance in the domains of their concerns. However, many of such advanced techniques may not be viable or practical when dealing with low-power IoT devices.

In this paper, we assume a situation wherein a trained model from a simple neural network architecture is given to low-power IoT devices. In general, IoT devices are limited in computing power, energy and memory capacity. Thus, running sophisticated and complex neural networks or advanced loss models are challenging [13], and even impractical for real-time IoT applications, such as self-driving cars. Additionally, changing the existing network model to a different one for better performance may take a significant amount of time, and it will delay the deployment stage. In general, shifting to a different network model requires several iterations of training (from a vast amount of data), testing and hyper-parameter tuning tasks. Thus, it is necessary to devise a low-complexity method to enhance the performance of deep learning models for IoT.

On the other hand, in the cases without IoT devices, there exist some sophisticated deep learning models that achieve a close-to-perfect accuracy. Nevertheless, it is impossible for a model not to make any mistakes, and such subtle errors may result in severe damage in mission-critical systems, such as battlefields and hospitals [23]. One may propose an application-specific way to further reduce errors, but it cannot be applied to general applications. Thus, it is necessary to devise a one-size-fits-all approach to assisting a deep learning model to achieve a better accuracy that can be used with general deep learning models.

To enhance the performance of a given deep learning model without incurring any additional significant or time-consuming computation, we propose a low-complexity novel framework which operates as an add-on to the general deep learning models without requiring any modification on the model’s side. The basic idea of the proposed framework is straightforward. For the given input x, the softmax output y is a vector of $y_{i}$’s, where $y_{i}=P\left(i|x\right)$ is the posterior probability of x belonging to class $i\in \{1,2,\cdots ,N_{c}\}$ and $N_{c}$ is the number of classes/categories. Although the following arg max operation takes the most likely class, it does not care about how close the corresponding probability is to 1. Furthermore, if the largest probability $y_{j}=\max\left\{y_{i}|y_{i}\in y\right\}$ was not so much different from the second-largest $y_{k}=\max\{y_{i}|y_{i}\in y\setminus \left\{y_{j}\right\}\}$, the model might be, what we call, *uncertain* about its prediction or decision. We propose to measure such *decision uncertainty* in a single quantity by using the well-known Jain’s fairness index [24], which has been widely used in the computer network domain [25]. In this paper, the computed fairness score of the softmax output is referred to as the *uncertainty score*, and is used to measure the level of uncertainty as to the model’s prediction.

In this paper, we propose a light-weight, uncertainty-score-based framework that effectively identifies incorrect decisions made by softmax decision-making models. We also propose a novel way to make mixed control decisions to enhance the target performance when the given deep learning model makes an incorrect decision. Additionally, the proposed framework does not make any change to the given trained model, but it simply puts an additional low-complexity function on top of the softmax classifier. The specific contributions we make in this work are summarized as follows:

- We propose a novel framework for the widely-used softmax decision-making models to enhance the performance of the given deep learning task without making any modification to the given trained model. Therefore, the proposed framework can be used with any neural network models using softmax loss.
- We propose to use an uncertainty score to gauge the level of uncertainty as to the model’s prediction. In a nutshell, the similarity among the softmax output is interpreted as how sure the model is about the current decision. To this end, we developed a practical method to effectively detect incorrect decisions to be made by the given deep learning model.
- We propose an effective way to enhance the performance of a deep leaning control system by making a mixed control decision. When the given model is believed to be yielding an incorrect decision/prediction, the proposed model replaces the model’s output with the probabilistic mixture of the available actions in order not to deviate much from the correct decision.
- We propose a low-complexity yet effective method to enhance the performance of the softmax decision-making models for low-power IoT devices. By using the time complexity terms, we show that the proposed framework does not incur any significant load from the given decision-making model, and thus, it can be used for online tasks.
- We show by an empirical study how the proposed framework effectively enhances the performance of the softmax decision-making tasks. To be specific, we carried out an experiment for IoT car control; we designed a control decision system that utilizes the softmax output to make a mixed, probabilistic car control decision when the model prediction is of low certainty.

Our work presented in this paper is innovative in that it suggests a new and systematic way of enhancing the performances of deep learning models. The proposed method treats the trained model as a *black box*, and thus, it can be applied to general deep learning models with little overhead. Additionally, it takes advantage of the *entire* softmax output to generate a decision when the model fails. The proposed approach is different from the previous studies focusing on either revising the deep neural networks or loss models. Additionally, by statistical and evaluation studies we show that not only the largest softmax output to be taken by the arg max operator, but also the actual values in the entire softmax output can be utilized to enhance the performances of deep learning models in the low-power IoT device control domain.

The rest of this paper is organized as follows. Section 2 introduces a brief overview on image classification and softmax loss. In the following Section 3, we describe the proposed framework to enhance the performances of deep neural networks with softmax loss. Section 4 presents experiment results, and the following Section 5 includes some discussion along with some notes as to the proposed framework. Finally, Section 6 concludes the paper.

## 2. Background and Motivation

In this section, we briefly review the classification problems on deep neural networks with softmax loss, and Jain’s fairness index, which plays a key role in the proposed framework as an uncertainty score. Then, we introduce our findings and understanding that motivated this work.

### 2.1. Background: Softmax and Uncertainty Score

As aforementioned, we consider ANN and CNN models with softmax loss for classification tasks. The lower layers in such networks can be seen as a feature extractor, and the last fully connected one as a classifier. As suggested in [18], we denote the combination of the cross-entropy loss and a softmax function of the last fully connected layer in a neural network by softmax loss. The given neural network is fed with the input data and trained using back-propagation by minimizing the loss which acts as an error signal. Therefore, which loss model to use is important for neural networks to effectively and efficiently train the network.

The classification problem, which frequently arises in deep learning tasks, is defined as follows: train the neural network model, $f:R^{d}\to \left\{1,\cdots ,N_{c}\right\}$ so that for the given input $x\in R^{d}$; let i=f(x) be the correct prediction on to which class does the input belong to. The last network layer outputs a vector y which is the set of probabilities that the given input belongs to each class. Taking the arg max operator on y yields the class indicator $i\in Z_{++}$, where the arg max operator finds the argument that yields the maximum value from a given function. Please note that depending on the beginning index of classes, we may have $i\in Z_{+}$ be the case. The softmax loss is defined as below, following similar notation to that used in [26]:

(1) $$L=-\frac{1}{M}\sum_{i=1}^{M}\log\frac{e^{W_{y_{i}}^{T}x_{i}+b_{y_{i}}}}{\sum_{j=1}^{N_{c}}e^{W_{j}^{T}x_{i}+b_{j}}}$$

where *M* is the size of the mini-batch, $x_{i}\in R^{l}$ is the learned feature that belongs to the $y_{i}$ class, *l* is the feature dimension, $W_{j}$ is the *j*-th column of the weight matrix W in the last fully connected layer and b is the bias.

One key component in the proposed framework is to use a fairness index or score to measure the level of uncertainty as to the model’s prediction, called the uncertainty score. We use the well-known Jain’s fairness index to compute the uncertainty score, which is defined as:

(2) $$J\left(y_{1},y_{2},\cdots ,y_{n}\right)=\frac{{\left(\sum_{i=1}^{n}y_{i}\right)}^{2}}{n\cdot \sum_{i=1}^{n}y_{i}^{2}}$$

where $y_{i}\in y$ and $y\in R^{n}$. The fairness index ranges from 1/n, representing the most unfair values among $y_{i}$’s, to 1 for being perfectly fair. Suppose a binary classification task, mapping each input to either of two classes. The softmax output vector y has two real values $y_{i}\in \left[0,1\right],\forall i=\left\{1,2\right\}$, and $\sum_{i}y_{i}=1$. For a given input $x_{1}$, if the softmax output is [0.0, 1.0], the model predicts the given input to belong to the second class. The corresponding fairness score is 0.5, having the worst fairness. Having a low fairness score implies that the model was certain about its decision, and thus, a high probability was given to the most-likely class. This is when there is little uncertainty in the model’s decision. For another input $x_{2}$, if the softmax output is [0.49, 0.51], the model predicts the given input to belong to the second class. The corresponding fairness score is 0.9996, achieving almost the perfect fairness. Having a high fairness score indicates that the model was uncertain about its decision, and thus, high probabilities were given to both classes. This is when there is a high uncertainty in the model’s decision.

For this reason, we interpret the fairness score among the softmax output as the uncertainty score which measures how uncertain the model is about its decision/prediction. The proposed framework relies much on the uncertainty score, and thus, the proposed framework is called UFrame hereafter.

### 2.2. Motivation: Uncertain Model Prediction

**Figure 1:**
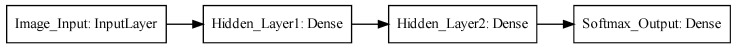
The simple ANN structure we used for the MNIST handwritten digit classification task. The second hidden layer is added to embed the learned features into 2D.

**Figure 2:**
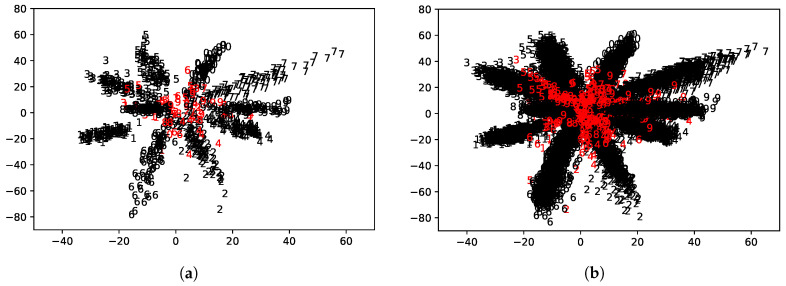
2D embedding of the learned features for the ANN is displayed. For better visibility, the left figure shows only the first 1000 samples in the test dataset. Correctly classified digits are colored in black, whereas misclassified ones are in red. (**a**) First 1000 samples in the test dataset; (**b**) entire sample of the test dataset.

To address what motivated our study, we first take the classification task for the MNIST handwritten digit database [27] as an example. We modeled a simple ANN, as shown in Figure 1, which yielded about 95% accuracy on the test data. Please note that we consider running the trained model on a low-power IoT device, and thus, we chose an extremely simple ANN model. We also obtained the 2D embedding result of the learned features, as shown in Figure 2, in which the misclassified digits are colored in red.

As can be seen from Figure 2, samples of the same labels form clusters, implying that different classes have different statistical characteristics. The misclassified samples tend to be apart from the corresponding cluster center. Said finding inspired many studies aiming at enlarging the inter-class gaps by using sophisticated loss models [17,18,19]. Since we assume a setting where re-training or adding additional complexity to the neural network model is challenging for the limited resources of IoT devices, we rather focus on discovering a *signal* of misclassification by means of the uncertainty score.

**Figure 3:**
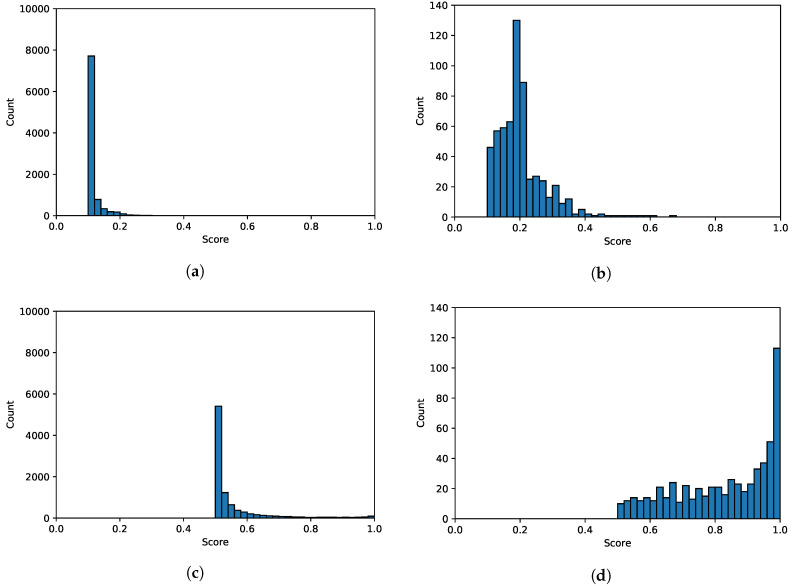
Histograms of the uncertainty scores with the interval of 0.02. Figures in the left column show the uncertainty score histograms of the correctly classified test samples, and the right column is for the misclassified test samples. (**a**) Uncertainty score histogram of the entire softmax output of the correctly classified samples. (**b**) Uncertainty score histogram of the entire softmax output of the misclassified samples. (**c**) Uncertainty score histogram of the two largest softmax outputs of the correctly classified samples. (**d**) Uncertainty score histogram of the two largest softmax outputs of the misclassified samples.

Figure 3 shows the uncertainty scores computed from the test dataset. The two upper images, Figure 3a,b, show the uncertainty score histogram of the softmax output of each test sample. On the other hand, the lower two images, Figure 3c,d, show the uncertainty score histogram of the two largest softmax outputs for each test sample. It is clear from both upper and lower figures that correctly classified samples have concentrated uncertainty distributions centering to the left, whereas misclassified samples have widespread distributions comparatively centering to the right. That is, most of the correct classifications are made with almost certainty; in other words, the softmax output of the likely digit is almost 1, and the rest are almost 0, resulting in a low uncertainty score. On the contrary, for the misclassified samples, the model prediction was uncertain, yielding high probabilities for many classes. In addition, by comparing Figure 3b–d, we can see that taking the two maximum softmax outputs to compute the uncertainty score, i.e., Figure 3d, results in a more distinct distribution pattern compared to its counterpart, Figure 3c.

The proposed UFrame is based off of the aforementioned findings that: (1) the uncertainty scores of the correctly classified samples have a distinct pattern compared to that of the misclassified ones; (2) the uncertainty scores of the two largest softmax outputs are more informative because the distribution of the correctly classified digits is concentrated to the left and that of the misclassified one is to the right, and the difference between those two is noticeable; and (3) different classes have different statistical characteristics. Such findings led to the proposed UFrame, which is explained in detail in the following section.

## 3. Proposed Method

Based on the findings in Section 2, we propose a novel framework, called UFrame, that effectively identifies misclassification and makes a mixed decision to enhance the performance of the given control task. By using a widely-used MNIST dataset, we validated UFrame as to whether it can effectively identify misclassified samples.

### 3.1. Algorithm Description

**Figure 4:**
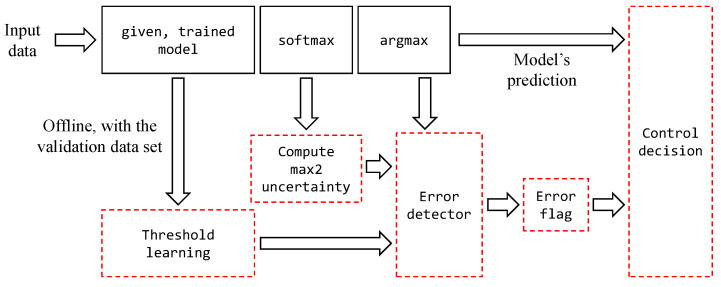
The proposed UFrame operates as a wrapper module for the given trained neural network model. UFrame includes the components inside the red dotted box, whereas the rest of the boxes in black solid lines belong the regular workflow of a general neural network model using softmax loss.

Figure 4 illustrates the proposed UFrame. Those boxes with black solid lines are the regular (deep) neural network workflow, and the ones with red dash lines belong to UFrame. The proposed UFrame runs as follows. First, by using the model output from the validation dataset, assuming it is available, it learns the error detection threshold which is to be explained in detail later. This step is carried out offline without disturbing the operation of the regular deep learning workflow. The computed threshold values get stored in a table for constant-time access. Then, for each new datum given to the model, UFrame takes the two largest values in the softmax output, and then computes the uncertainty score. Said score is referred to as the *max2 uncertainty* score hereafter. The error detector module compares the max2 uncertainty score to the threshold to determine whether or not the upcoming decision by the arg max function is likely to be incorrect. If the max2 uncertainty score exceeds the threshold, the error flag is set to true, 1 or on; otherwise, it sets the flag to false, 0 or off. If the error flag is false, the control decision module uses the model’s prediction as it is. If the error flag is true, on the other hand, the control decision module makes a mixed control decision as follows.

Let *i* be a class index, indicating one of the possible actions the IoT device can make. It can be one of the directions an IoT car can move towards, or a robot arm movement. In the regular deep learning workflow, for an input datum, the deep learning model predicts which action to take, and passes the decision to the IoT device as a control signal. Having $N_{c}$ number of classes implies there are the same number of actions available. Let each viable action be $u_{i}$ with *i* being the class index. The deep learning model predicts to which class *i* the input belongs to, and the corresponding control decision becomes $u_{i}$, if the error flag is off. That is, the model’s prediction is passed to the IoT device as it is when the error flag is off. However, if the error flag is on, the model’s prediction is uncertain, and thus, the control decision module makes a mixed control decision in the following manner. It first transforms all possible actions $u_{i},\forall i$ to $N_{c}$ number of different unit vectors in an $R^{q}$ space, where *q* is task-specific. Given the softmax output y, the mixed control decision becomes $\sum_{i=1}^{N_{c}}y_{i}\cdot u_{i}$, where $y_{i}\in y$.

The error detection threshold plays a key role in UFrame; it is used to determine whether or not the upcoming decision for the input data is likely to be incorrect. The threshold learning algorithm (TLA) is carried out offline as follows. Given the validation dataset, TLA feeds the validation dataset to the trained model to retrieve the softmax output of each sample therein. Here, it does not matter whether or not the validation dataset is the same dataset that was used when training the model. At the same time, for each sample TLA sets a binary flag indicating whether the corresponding sample has been correctly identified, and stores the true label indicating the class to which each sample belongs. With the acquired records of (softmax output vector, binary flag, true label) for each sample, TLA computes and collects the max2 uncertainty scores among the correctly classified samples for each class. Please note that having a different threshold for each class has shown better performance from our empirical study (see Section 3.2). Assuming the distribution of max2 uncertainty score follows a (one-sided) normal distribution within each class (see Figure 3), UFrame computes the mean and standard deviation of the max2 uncertainty scores among the correctly classified samples.

As for the last step, TLA takes in a design parameter $\alpha \in Z_{++}$ so that the detection threshold for class *i* is $th_{i}=m_{i}+\alpha \cdot stdev_{i}$, where $m_{i}$ is the mean of the max2 uncertainty scores of class *i* and $stdev_{i}$ is the standard deviation. The detection threshold for class *i* is referred to as $th_{i}$ hereafter. The parameter α controls how *rigid* the error detector is to be, which will be explained in Section 3.2.

### 3.2. Validation: Identifying Misclassified Samples

To validate the performance of UFrame as to whether it can correctly identify the misclassified samples, we have applied UFrame to the MNIST digit recognition task. The MNIST database includes 60,000 samples for training, and 10,000 samples for testing. We further divided the training dataset into 50,000 for training and 10,000 for validation. We have used a similar ANN as in Figure 1, except the 2D embedding layer, which was omitted here. After tuning hyper-parameters, the neural network achieved 97% accuracy on the test dataset.

The per-class mean and standard deviation of max2 uncertainty score are shown in Table 1, and they indeed are different from each other. As aforementioned, difference classes have different characteristics, and having a different threshold for each class has shown better detection performance. Given the per-class mean and standard deviation values, we can compute the error detection threshold for class *i* as $th_{i}=m_{i}+\alpha \cdot stdev_{i}$, where i∈{0,1,⋯,9} for the given 10-digit recognition task. Please note that the threshold is computed from the correctly identified samples in the validation dataset, while the performance of UFrame is measured on the test dataset. When the neural network model processes an input datum x, UFrame intercepts the softmax output and computes the max2 uncertainty score. The error detector then compares the max2 uncertainty score to $th_{i}$, where *i* is the model’s prediction. If the max2 uncertainty score exceeds $th_{i}$, the error flag is set to true, and false if not.

**Figure 5:**
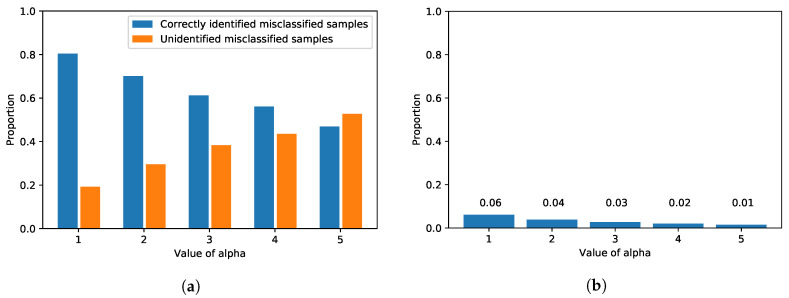
The detection performance of UFrame as to the MNIST test dataset is shown. (**a**) Among the samples misclassified by the ANN model, the proportions of the correctly identified samples (i.e., error flag was true) and those that were not (i.e., error flag was false) are shown in blue and orange, respectively. (**b**) Among the correctly identified samples by the ANN model, the proportion of the incorrectly identified samples (i.e., error flag was true) is shown with the corresponding value.

Figure 5 shows the performance of UFrame in terms of error detection, and the exact values are reported in Table 2. In the figure, the x-axis is the value of α for the threshold, and the y-axis is the proportion of the corresponding samples. Regardless of α, the number of misclassified test samples from the ANN model remains the same. Among those misclassified samples by the ANN model, the proportion of the ones that are identified and reported by UFrame is shown in blue bars in Figure 5a. A smaller value of α makes the error detector more rigid or strict, and thus, UFrame frequently encounters samples violating the threshold. As a result, with α=1, UFrame successfully identified 218 cases of misclassification out of 271 in the test dataset, which is about 80% of the misclassified samples. On the other hand, a larger α increases the threshold for each class. Thus, UFrame becomes more tolerant to the samples with high max2 uncertainty score, resulting in a smaller number of cases with the error flag being true. As a reminder, high max2 uncertainty score means the first and second larges values in the softmax output are similar to each other. That is, the model is uncertain about its prediction, and thus, it is likely to be incorrect.

However, the value of α has a negative effect at the same time. Figure 5b shows the proportion of the samples detected by the error detector among the correctly identified samples. As aforementioned, since α determines how strict or tolerant the error detector will become, a smaller α results in more *false positives*, i.e., an error flag is on even for the correctly classified samples. However, such cases amount to only a little portion—at most 6%. Additionally, such false positives indeed have a negligible effect on the control decision in terms of both time complexity and decision accuracy. Although UFrame will decide to make a mixed control decision instead of using the model’s prediction, since the correct class was given the largest softmax output, the mixed decision will be biased much to the correct decision.

## 4. Experiments

**Figure 6:**
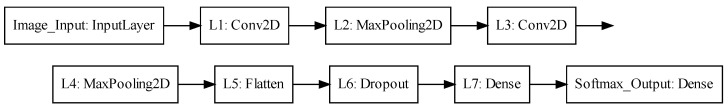
The displayed CNN model was used for the indoor self-driving task.

In order to validate the performance of UFrame with a real-world IoT application, we have carried out an experiment. Please note that the use of the proposed framework is not limited to IoT devices. It can also be used for general-purpose and programmable low-power sensor devices. The considered use case here is making control decisions for indoor self-driving toy cars. For the low computing power of single board computers (SBC) such as Raspberry Pi (RPi), we simplified the self-driving task to image classification. At the beginning, a series of manual driving tasks were carried out by a human, during which images through the USB camera and the human controller’s input key strokes, i.e., left, forward and right, are collected. The acquired image dataset was then increased by flipping horizontally and shifting by a small amount of pixels. The entire dataset was divided into three, i.e., training (42,446 samples), validation (5305 samples) and testing (5306 samples), before training the CNN model (see Figure 6). To speed up the real-time control decision-making process, the trained model to run on RPi was converted to a TensorFlow Lite equivalent. The trained model was an image classifier that mapped the incoming camera image into one of the three different classes indicating steering wheel directions, i.e., left, forward or right.

**Figure 7:**
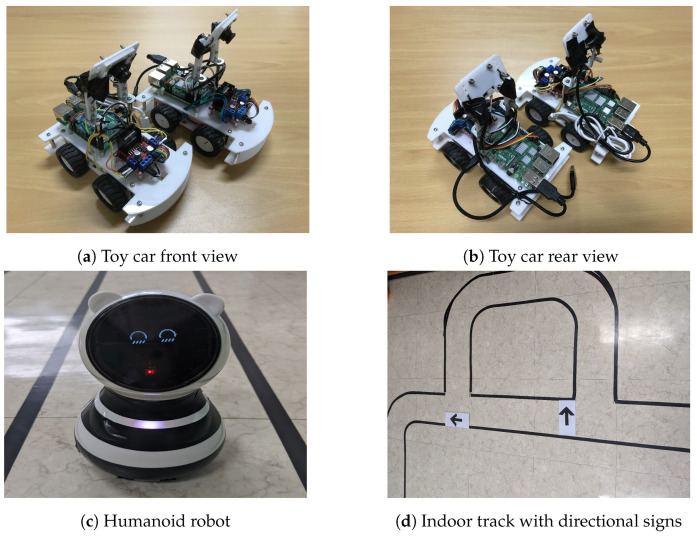
Toy cars with Raspberry Pi v4 and an L298N motor drive shield, a humanoid robot, were used for the experiments on an indoor track with directional signs.

In fact, the self-driving task can be implemented without deep learning in many cases. For example, an IoT car can detect the lanes on both sides with a feature extraction technique, e.g., Hough transform [28]. Then, by comparing the centers of the lanes on both sides and the center of the car, an IoT car can drive autonomously. However, in this experiment, we considered a realistic situation where the drive or journey could be interrupted by other moving objects, such as humanoid robots, as in Figure 7c. Additionally, there are several intersections, and an IoT car can decide on which direction to go by the directional signs (see Figure 7d). For a self-driving car to successfully drive while complying with the simple rules of the road, i.e., following the directional signs and stopping when blocked by other objects, we chose to solve the self-driving task by CNN-based deep learning approach.

Each toy car shown in Figure 7 carried an RPi v4 as a controller and an L298N motor drive shield on its back. The RPi was powered by a battery, which is invisible in the figure, with 5.0 V and 2.0 A output. The IoT toy cars were connected via a built-in WiFi interface so that they could communicate with each other and with the road side unit (RSU). The RSU broadcast heavy-traffic and accident information, and a toy car receiving such information was to slow down. Toy cars could return to the normal speed only when another message indicating the clearance of the situation was received from RSU. If a car failed to receive any information from the RSU, the car which successfully received the information could forward it to other cars nearby. Please note that such reception failure can happen for many reasons, such as out of the transmission range of RSU, packet collision and packet drop, to name a few.

**Figure 8:**
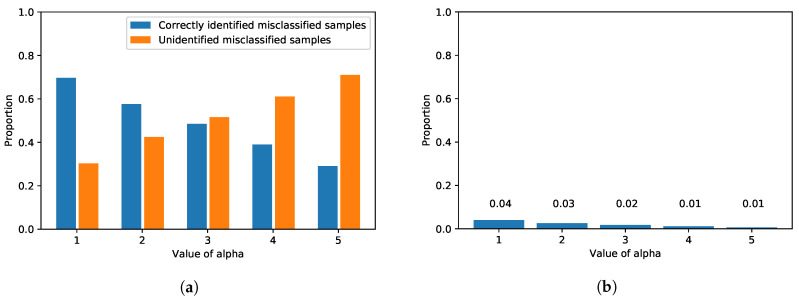
The detection performance of UFrame as to the IoT car image dataset is shown. (**a**) Among the misclassified samples by the CNN model, the proportions of the correctly identified samples (i.e., error flag was true) and those were are not (i.e., error flag was false) are shown in blue and orange, respectively. (**b**) Among the samples correctly identified by the CNN model, the proportion of the incorrectly identified samples (i.e., error flag was true) is shown with the corresponding value.

Through a series of trainings with different configurations, five epochs with the mini-batch size of 128 were chosen to avoid over-fitting. The resulting model yielded an accuracy of about 95% on the test dataset. Again, we measured the max2 uncertainty score of the softmax output from the correctly classified samples in the validation dataset. The per-class means and standard deviations of the max2 uncertainty scores are reported in Table 3. We also evaluated the detection performance on the IoT car image dataset, and the results are shown in Figure 8. Please note that the same evaluation was carried out for the MNIST dataset as well (see Figure 5). It is clear from both figures that, although one dataset along with its application is very much different from the other in terms of the underlying neural network architecture, the number of classes and the contents in the images, the proposed framework can effectively identify misclassified samples in both applications (see Figure 5a and Figure 8a), and the proportion of the incorrectly identified samples is insignificant overall (see Figure 5b and Figure 8b). Although there is a difference in the patterns between Figure 5a and Figure 8a—the orange bar exceeds the blue bar with a lesser value of α in the IoT car dataset than in the MNIST dataset—that was only because of the different number of classes and the samples in each neural network and dataset, respectively.

The misprediction of the model can be regarded as a steering wheel direction error by greater than or equal to 90 degrees. If the model misses a forward direction, for example, the wheel direction error is exactly 90 degrees no matter which direction the model mistakenly chooses. If the model misses a left direction, for example, the wheel direction error is either 90 or 180 degrees for mistakenly choosing forward or right direction, respectively.

**Figure 9:**
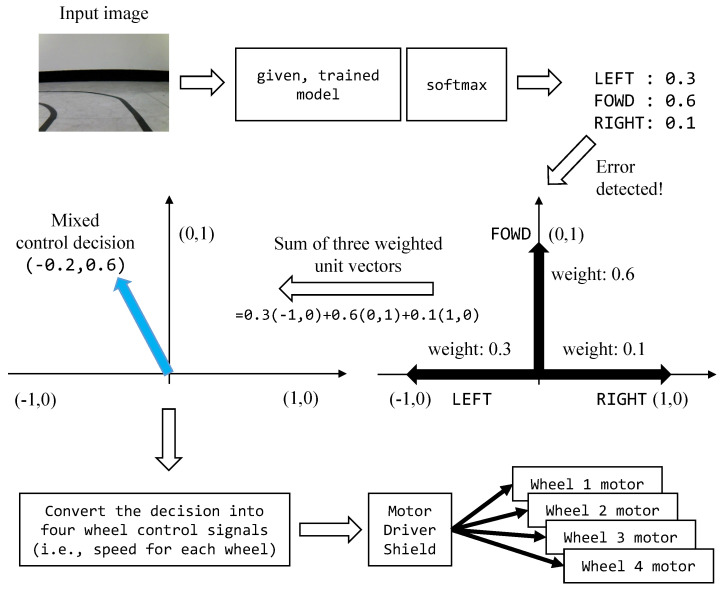
The way that UFrame makes a mixed control decision when the max2 uncertainty score exceeds the threshold.

For this steering wheel control task on an IoT car, when the model’s prediction is uncertain, the proposed UFrame can make a mixed control decision in the following manner by levering the decision uncertainties, i.e., the softmax output. If the max2 uncertainty score of the softmax output is below the threshold of the class to which the model classified the current input, the model prediction is passed to the toy car an the control input as it is. On the other hand, if the uncertainty score exceeds the threshold, the control output of the car, i.e., the steering direction, becomes a probabilistic combination of the three directions as shown in Figure 9. Suppose the case described in the figure: for the given input image, the softmax output is [0.3, 0.6, 0.1] for left, forward and right directions. Additionally, suppose the max2 uncertainty score has exceeded the threshold. Then, instead of using the model prediction (i.e., moving straight since 0.6 is the largest among the softmax output) to steer the toy car, UFrame makes a mixed control decision as follows. Let [−1,0], [0,1] and [1,0] be the unit vectors representing left, forward and right directions, respectively. Additionally, let each softmax output be the probability of the given input image belonging to the corresponding direction. UFrame multiplies a unit vector and the corresponding probability for each direction, and then adds them together to produce a vector $\left[v_{x},v_{y}\right]$. The $v_{x}$ indicates the normalized velocity towards left or right depending on the sign (+ or −). Likewise, $v_{y}$ indicates the normalized velocity towards the forward direction. The next step is to convert $\left[v_{x},v_{y}\right]$ to the motor speed for the four wheels which will be passed to the corresponding motors via the motor driver shield.

To evaluate and compare the performance of UFrame, we have measured the errors in angle, i.e., the angle difference between the correct direction and the direction chosen by either the CNN model or UFrame (i.e., mixed control decision) for each sample in the test dataset. Please note that when the CNN model makes an incorrect decision on direction, the error in angle amounts to at least 90 degrees. The performance evaluation result with respect to the different values of α is shown in Table 4.

The CNN model which has nothing to do with α made correct decisions on 5058 test samples, and missed 248, resulting in about 95% accuracy on the test dataset. Those misses deviate from the correct steering angle by at least 90 degrees. On the other hand, for any values of α, UFrame made only eight misses of such large-degree mistakes. As aforementioned, smaller values of α make the error detector more strict. In the case of α=1, for example, UFrame did not set the error flag only for 4636 samples, which is the smallest. On the other hand, in the case of α=5, 5014 samples resulted in taking the model’s decision as it was without making a mixed control decision due to the large threshold. As α decreases, UFrame makes more mixed control decisions, and results in the smallest the number of control mistakes with 50+ degrees of angle differences to the correct angle. That proves that making a mixed control even in the case wherein the model makes a correct prediction does not degrade the quality of the decision, since in such cases the softmax output is biased to the correct decision and so does the mixed decision. On the contrary, as α increases, UFrame makes less mixed control decisions, but suffers from having many control mistakes with the same amount of angle errors (i.e., >50 degrees). However, for any values of α, UFrame outperforms the CNN model.

The CNN model produced 248 errors, or in other words, the model misclassified 248 input images. However, due to the high accuracy of the model (i.e., about 95%), even when the model made an incorrect prediction, the softmax output for the correct direction was still large, increasing the uncertainty score. The high uncertainty score lets the proposed framework make a mixed decision. By mixing the three unit vectors with the softmax output being the weight, the mixed control decision leans towards the correct decision. This is why the proposed framework makes a much smaller number of errors than the CNN model. On the other hand, regardless of α, the proposed framework produced eight cases with large angle errors, i.e., ≥90 degrees. This happens when the softmax output of the model is completely incorrect, giving almost zero probability to the correct direction. When the majority of the probability is given to one of the incorrect directions, the error flag becomes off, preventing the proposed framework from making a mixed decision. When the other two incorrect decisions receive a similar amount of probability and the correct direction is either left or right, the proposed framework makes a mixed decision, but it deviates much from the correct decision. Such cases happened for the input images that did not have any meaningful information for the CNN model to predict which direction to go—for example, images having no track/lane at all and blurry images (from camera shake).

## 5. Discussion and Notes

### 5.1. Possible Variation on the Proposed Framework

One possible variation of making a mixed control decision is using stochastic sampling which is well-known in generative deep learning [29]. To be specific, when the max2 uncertainty score exceeds the threshold, UFrame can randomly sample the final output, e.g., model decision or prediction, from the distribution specified by the softmax output. Although it is a viable strategy, it may result in making large errors in the case of the IoT car control, for example, due to the randomness. The proposed mixed control decision module also makes a decision in a probabilistic manner, but the resulting decision is biased according to the model’s prediction. Therefore, as long as the model has learned features well enough and results in a high accuracy on the test dataset, the proposed mixed control decision model will outperform the stochastic-sampling-based approach.

### 5.2. Working with Advanced Softmax Losses

The proposed UFrame does not require any changes when used with different softmax loss models. The advantages of the advanced softmax losses only contribute to enhancing the performance of the proposed UFrame. Sophisticated softmax loss models tend to maximize the inter-class distances while minimizing the intra-class distances on a given metric space. Therefore, the number of incorrect decisions, i.e., misclassifications—our interest, is expected to be reduced. Additionally, due to the enhanced capability of discriminating input data belonging to one class from others, the uncertainty score distribution of the correctly classified input is expected to be better distinguishable from those of the misclassified ones.

### 5.3. Complexity of the Proposed Framework

The proposed UFrame incurs the following two different types of complexity. One is the time complexity caused by TLA. TLA feeds the entire validation dataset to the given, trained model, and thus, the time complexity for threshold learning depends on the complexity of the model and the number of validation samples. However, since TLA runs offline, it does not slow down the real-time performance of the model operating with UFrame. On the other hand, the other operations of UFrame, i.e., computing the max2 uncertainty, setting the error flag and making a mixed control, may delay the model to some degree. It takes $O(N_{c})$ and O(1) to compute the max2 uncertainty and to determine whether or not to set the error flag, respectively. In the case of making a mixed control decision, UFrame needs an extra $O(N_{c})$ time. Overall, UFrame adds a time complexity of $O(N_{c})$ to the entire decision-making process, which is negligible.

### 5.4. Performance of the Proposed Framework in General

From the evaluation studies presented in both Section 3 for the MNIST image and Section 4 for the IoT car image dataset, we have shown the effectiveness of the proposed framework in different applications. To be specific, for a small value of α the proposed framework can identify the majority of the misclassified samples from the deep learning model by using the threshold-based error detection algorithm. Considering the large difference in the two datasets and in the corresponding applications (see Section 4 for our discussion on how different one set is from the other), the results we have presented in the previous two sections suggest that the proposed framework can be applied to a wide-range of applications using softmax decision-making models, which is why we have considered two very different applications in this work.

However, the performance of the error detector depends on the accuracy of the underlying deep learning model. As aforementioned, the threshold learning algorithm uses the correctly classified samples from the model. A poor accuracy of a model will result in a relatively small number of correctly identified samples with a high uncertainty score. Then, the learned threshold can easily be biased, degrading the performance of the error detector for having an incorrect threshold. Therefore, the proposed framework should be used with a model which guarantees a high level of classification accuracy. The same applies to the control decision algorithm in the proposed framework.

## 6. Conclusions

In this paper, we have proposed an effective framework that enhances the performance of softmax decision-making deep learning models. Inspired by the idea that the uncertainty score of the softmax output indicates how uncertain the model is as to its decision, the proposed uncertainty-score-based framework effectively identifies the majority of the misclassified or misrecognized samples. In other words, the proposed framework makes use of the distribution of the probabilities or uncertainties in the softmax output to discover incorrect decisions made by the model. In addition, we showed by an empirical study how to effectively enhance the performance of the given, trained model by making a mixed control decision when the model’s output was likely to be incorrect. Additionally, the proposed UFrame does not make any modification to or put any computationally heavy burden on the existing model.

Due to being low in complexity and compatible with general softmax-based deep learning models, the proposed framework can boost the field of deep learning with IoT. Additionally, the proposed algorithm that makes a mixed control decision can be applied to the fields wherein precise control decisions are required, especially for robots. The capability of the proposed framework is not limited to IoT or general-purpose sensors, and thus, it can be used for enhancing the performances of sophisticated deep learning models for classification/recognition by identifying incorrectly classified samples. We envision that the proposed framework will play an important role in mission-critical applications with or without IoT, where the tolerable error rate is strictly limited.

As for the future work, we plan to deploy the proposed framework on various IoT devices with different deep learning applications to evaluate the performance of the proposed framework with respect to the computing capacities of IoT devices, the complexity of the deep learning model and its classification/recognition performance. We also plan to carry out studies on performance comparisons between the proposed framework and other similar approaches in terms of time/space complexity and accuracy (or error rate) for classification/recognition or control decisions. We also plan to extend our work to solve difficult control tasks wherein the decision domain is continuous and wherein the control decisions are affected by external random factors.

## Figures and Tables

**Table 1:** Per-class mean and standard deviation of the max2 uncertainty scores among the correctly classified samples, acquired from feeding the validation dataset.

Digit (i.e., Class)	0	1	2	3	4	5	6	7	8	9
mean	0.506	0.521	0.519	0.527	0.520	0.523	0.514	0.516	0.515	0.519
standard deviation	0.039	0.064	0.068	0.080	0.068	0.073	0.055	0.057	0.060	0.066

**Table 2:** The exact values reported in Figure 5 when there are 10,000 test samples.

Value of α	1	2	3	4	5
Misclassification by ANN	271	271	271	271	271
Misclassification with error flag on	218	190	166	152	127
Misclassification with error flag off	52	80	104	118	143
Correct classification with error flag on	589	376	265	196	138

**Table 3:** Per-class means and standard deviations of the max2 uncertainty score acquired from feeding the validation dataset to the trained model.

Key Stroke or Direction (i.e., Class)	Left	Forward	Right
mean	0.530	0.547	0.520
standard deviation	0.085	0.097	0.066

**Table 4:** Performance evaluation results: for different values of α, the number of samples with respect to the angle difference between the correct direction and the chosen direction by the CNN model and UFrame is shown below. The number of samples in the test dataset was 5306.

Model	UFrame					CNN
Value of α	1	2	3	4	5	n/a
no difference	4636	4775	4860	4925	5014	5058
≤10 degrees	104	23	9	6	4	0
>10 degrees	566	508	437	375	288	0
>20 degrees	419	419	415	367	287	0
>30 degrees	337	337	337	337	273	0
>40 degrees	270	270	270	270	255	0
>50 degrees	228	228	228	228	236	0
>60 degrees	184	184	184	186	218	0
>70 degrees	143	143	145	173	213	0
>80 degrees	91	104	132	171	213	0
≥90 degrees	8	8	8	8	8	248

## Abbreviations

The following abbreviations are used in this manuscript:

ANN	Artificial Neural Network
CNN	Convolutional Neural Network
IoT	Internet of Things
